# Blood MMP-9 measured at 2 years after lung transplantation as a prognostic biomarker of chronic lung allograft dysfunction

**DOI:** 10.1186/s12931-024-02707-3

**Published:** 2024-02-09

**Authors:** Adrien Tissot, Eugénie Durand, Thomas Goronflot, Benjamin Coiffard, Benjamin Renaud-Picard, Antoine Roux, Xavier Demant, Jean-François Mornex, Loïc Falque, Mathilde Salpin, Jérôme Le Pavec, Thomas Villeneuve, Véronique Boussaud, Christiane Knoop, Antoine Magnan, David Lair, Laureline Berthelot, Richard Danger, Sophie Brouard, the COLT consortium, Elodie Blanchard, Xavier Demant, Virginie Hulo, Maria Ruiz-Patino, Maarten Vander Kuylen, Youri Sokolow, Constantin Stefanidis, Isabelle Huybrechts, Laurent Perrin, Fabio Taccone, Isabelle Etienne, Christiane Knoop, Anna Roussoulières, Maya Hites, Agnes Lambert, Axelle Hemelsoet, Pierrick Bedouch, Amandine Briault, Loic Falque, Quentin Perrier, Christel Saint Raymond, Samarmar Chacaroun, Yoann Gioria, Joane Quentin, Renaud Grima, Gabrielle Drevet, Jean-Michel Maury, François Tronc, Philippe Portan, Jean-François Mornex, Claire Merveilleux Du Vignaud, Eva Chatron, Jean Charles Glérant, Ségolène Turquier, Salim Si Mohamed, Vincent Cottin, Lara Chalabresse, Chantal Dubois, Aurélie Rea, Médéric Reignier, Julia Canterini, Nicolas Carlier, Véronique Boussaud, Romain Guillemain, Xavier-Benoit D’Journo, Pascale-Alexandre Thomas, Delphine Trousse, Geoffrey Brioude, David Boulate, Alex Fourdrain, Fabienne Bregeon, Stéphane Delliaux, Martine Reynaud-Gaubert, Bérengère Coltey, Nadine Dufeu, Benjamin Coiffard, Julien Bermudez, Ana Nieves, Hervé Dutau, Julie Tronchetti, Jean-Yves Gaubert, Paul Habert, Mathieu Di Biscéglie, Agnes Basire, Pascal Pedini, Florence Daviet, Christophe Guervilly, Sami Hraiech, Jean Marie Forel, Louis Delamarre, Aude Charvet, Ines Gragueb-Chatti, Pierre Mora, Daniel Laurent, Sophie Giusiano, Jean-Philippe Dales, Mélanie Gaubert, Marc Laine, Philippe Lacoste, Christian Perigaud, Jean-Christian Roussel, Thomas Senage, Antoine Mugniot, Isabelle Danner, Adrien Tissot, Charlotte Bry, Morgane Penhouet, Emmanuelle Eschapasse, Delphine Horeau-Langlard, François-Xavier Blanc, Thierry Lepoivre, Mickael Vourch, Sophie Brouard, Richard Danger, Megguy Bernard, Elodie Godard, Régine Valéro, Karine Maugendre, Eugénie Durand, Nataliya Yeremenko, Aurore Foureau, David Lair, Géraldine Gallot, Mathilde Berthome, Jérôme Le Pavec, Gaëlle Dauriat, Pauline Pradere, Séverine Feuillet, Samuel Dolidon, Chahine Medraoui, Pierre Gazengel, Adrian Crutu, Amir Hanna, Elie Fabre, Olaf Mercier, Delphine Mitilian, Justin Issard, Dominique Fabre, Yves Castier, Pierre Mordant, Pierre Cerceau, Antoine Girault, Arnaud Roussel, Enora Atchade-Thierry, Sylvain Jean-Baptiste, Sandrine Boudinet, Sébastien Tanaka, Aurélie Gouel, Philippe Montravers, Nathalie Zappella, Aurélie Snauwaert, Parvine Tashk, Brice Lortat-Jacob, Tiphaine Goletto, Domitille Mouren, Lise Morer, Mathilde Salpin, Hervé Mal, Armelle Marceau, Gaëlle Weisenburger, Vincent Bunel, Adèle Sandot, Kinan El Husseini, Pierre Halitim, Lucie Genet, Sabrina Trigueiros, Alice Savary, Hakima Rabia, Pierre-Emmanuel Falcoz, Anne Olland, Charlotte Ponte, Charles Tacquard, Garib Ajob, Olivier Collange, Antoine Pons, Xavier Delabranche, Olivier Helms, Anne Roche, Benjamin Renaud-Picard, Romain Kessler, Tristan Degot, Sandrine Hirschi, Armelle Schuller, Anne Dory, Florence Toti, Nadia Benkirane-Jessel, Laurence Kessler, Julien Stauder, Edouard Sage, Francois Parquin, Sandra De Miranda, Clément Picard, Antoine Roux, Olivier Brugière, Béatrice D’Urso, Marc Stern, Akounach Mbarka, Antoine Magnan, Quentin Marquant, Isabelle Schwartz, Helene Salvator, Tiffany Pascreau, Thomas Villeneuve, Marion Dupuis, Marlène Murris-Espin, Pierre Rabinel, Laurent Brouchet, Laure Crognier, Olivier Mathe, Frédérique Legenne, Myriam Barthes, Blandine Vilquin, Anne-Laure Costes, Isabelle Recoche, Anne Bergeron, Gregory Berra, Angela Koutsokera

**Affiliations:** 1 CHU Nantes, INSERM, Service de Pneumologie, l’institut du thorax, Center for Research in Transplantation and Translational Immunology (CR2TI), UMR 1064, Nantes Université, 44093 Nantes, France; 2 CHU de Nantes, Inserm, Centre de Recherche Translationnelle en Transplantation et Immunologie (CR2TI), Nantes Université, Nantes, France; 3 CHU Nantes, Pôle Hospitalo-Universitaire 11: Santé Publique, Clinique des données, INSERM, CIC 1413, Nantes Université, Nantes, France; 4 Department of Respiratory Medicine and Lung Transplantation, APHM, Hôpital Nord, Aix Marseille Univ, Marseille, France; 5 Department of Respiratory Medicine and Strasbourg Lung Transplant Program, Inserm UMR 1260, Université de Strasbourg, Hôpitaux Universitaires de Strasbourg, Strasbourg, France; 6 Pneumology, Adult Cystic Fibrosis Center and Lung Transplantation Department Hôpital Foch, Suresnes, INRAe UMR 0892, Paris Transplant Group, Université de Versailles Saint Quentin Paris-Saclay, Paris, France; 7 Service de Pneumologie, Centre Hospitalier Universitaire de Bordeaux, Pessac, France; 8 Université Lyon 1, PSL, EPHE, INRAE, IVPC, Hospices Civils de Lyon, groupement hospitalier est, service de pneumologie, Orphalung, RESPIFIL, Université de Lyon, Lyon, France; 9 Service Hospitalier Universitaire de Pneumologie et Physiologie, CHU Grenoble Alpes, Pôle Thorax et Vaisseaux, Grenoble, France; 10 APHP Nord-Université Paris Cité, Hôpital Bichat, Service de Pneumologie B et Transplantation Pulmonaire, PHERE UMRS 1152, Université Paris Cité, Paris, France; 11 Service de Pneumologie et Transplantation Pulmonaire, Groupe hospitalier Marie-Lannelongue -Saint Joseph, Le Plessis-Robinson, Université Paris-Saclay, UMR_S 999, INSERM, Université Paris-Sud, Le Kremlin Bicêtre, France; 12 CHU Toulouse, Service de Pneumologie, Université Toulouse III-Paul Sabatier, Toulouse, France; 13 APHP, Service de Pneumologie, Hôpital Cochin, Paris, France; 14 Service de Pneumologie, CHU Erasme, Brussels, Belgium; 15 CHU Nantes, Nantes Université, Institut du Thorax, Lung O2, Nantes, France

## Abstract

**Background:**

Long-term outcomes of lung transplantation (LTx) remain hampered by chronic lung allograft dysfunction (CLAD). Matrix metalloproteinase 9 (MMP-9) is a secretory endopeptidase identified as a key mediator in fibrosis processes associated with CLAD. The objective of this study was to investigate whether plasma MMP9 levels may be prognostic of CLAD development.

**Methods:**

Participants were selected from the Cohort in Lung Transplantation (COLT) for which a biocollection was associated. We considered two time points, year 1 (Y1) and year 2 (Y2) post-transplantation, for plasma MMP-9 measurements. We analysed stable recipients at those time points, comparing those who would develop a CLAD within the 2 years following the measurement to those who would remain stable 2 years after.

**Results:**

MMP-9 levels at Y1 were not significantly different between the CLAD and stable groups (230 ng/ml vs. 160 ng/ml, p = 0.4). For the Y2 analysis, 129 recipients were included, of whom 50 developed CLAD within 2 years and 79 remained stable within 2 years. MMP-9 plasma median concentrations were higher in recipients who then developed CLAD than in the stable group (230 ng/ml vs. 118 ng/ml, p = 0.003). In the multivariate analysis, the Y2 MMP-9 level was independently associated with CLAD, with an average increase of 150 ng/ml (95% CI [0–253], p = 0.05) compared to that in the stable group. The Y2 ROC curve revealed a discriminating capacity of blood MMP-9 with an area under the curve of 66%.

**Conclusion:**

Plasmatic MMP-9 levels measured 2 years after lung transplantation have prognostic value for CLAD.

**Supplementary Information:**

The online version contains supplementary material available at 10.1186/s12931-024-02707-3.

## Introduction

Lung transplantation (LTx) has the potential to bring survival benefits and improve quality of life in selected candidates [1, 2]. However, long-term outcomes remain low, with an overall survival of 63% at 5 years post-transplantation [3]. The main long-term limitation is chronic lung allograft dysfunction (CLAD), which is responsible for most deaths after the first posttransplant years [4]. A large part of CLAD physiopathology remains unclear, but significant advances have been made to better understand the two clinically and functionally well-defined phenotypes of CLAD: bronchiolitis obliterans syndrome (BOS) and restrictive allograft syndrome (RAS). Briefly, repeated injuries of the airway epithelium, alveoli or lung endothelial cells result in chronic inflammation, wound healing, recruitment and proliferation of fibroblasts and aberrant deposition of extracellular matrix, leading to small airway fibrotic obliteration in BOS and alveolar fibroelastosis in RAS [5]. CLAD risk stratification of LTx recipients is presently an unmet need. A multisystemic approach is certainly the way forward in a highly complex situation in which immunology, medications, environment and patient’s behaviours have an important impact on outcomes. Matrix metalloproteinases (MMPs) are a family of zinc-dependent endopeptidases involved in the degradation of various proteins of the extracellular matrix. MMPs play a role in tissue remodelling during various physiological processes, such as angiogenesis, embryogenesis, morphogenesis and wound repair, as well as in pathological conditions, such as fibrotic disorders and cancer. Matrix metalloproteinase 9 (MMP-9) is part of the gelatinase subfamily, which has a distinct collagen-binding domain. It can be secreted by a variety of cells, including epithelial cells, fibroblasts, macrophages and T cells [6]. MMP-9 has been identified as a key mediator in processes associated with CLAD, such as extracellular matrix reorganization, cell migration, epithelial to mesenchymal transition and immune response [7]. Interestingly, MMP-9 is an old suspect in BOS, and several works have found increased gelatinolytic activity and/or MMP-9 concentrations in bronchoalveolar lavage (BAL) from recipients with BOS [8–11]. Two studies reported that almost 6 months before the diagnosis of BOS, increased BAL MMP-9 activity was observed [12, 13]. However, the relevance of MMP-9 in peripheral blood remains debated, whereas the clinical need for minimally invasive biomarkers of CLAD is high [14]. Previous data from our group showed that blood MMP-9 levels may be higher in CLAD recipients [15]. To evaluate CLAD risk factors that could enable preventive intervention, we considered the following time points of analysis: 1 and 2 years post-transplantation. Our objective in this study was to validate whether plasma MMP-9 levels at 1 year and 2 years post-transplantation can predict future CLAD onset.

## Materials and methods

### Participants

Lung transplant recipients (LTRs) were recruited within the multicentre cohort in lung transplantation [COLT], NCT00980967) study (Comité de Protection des Personnes Ouest 1-Tours, 2009-A00036-51). The study was approved by the local ethical committee, and all participants provided written informed consent. The COLT study protocol has been previously described (Additional file 1: S1) [16]. Briefly, participants were included in the cohort before transplantation. Planned visits were at the time of transplantation and then every 6 months up to 5 years post-transplantation. Blood samples were taken at each visit. This study was conducted in accordance with the Declaration of Helsinki and complies with the International Society for Heart and Lung Transplantation statement on Transplant Ethics.

### Clinical phenotype

All patients within the COLT study underwent individual phenotyping by an adjudication committee that gathered at least 5 investigator physicians from the different participating centres. Pulmonary function tests, relevant chest computed tomography and medical history, especially potential confounding factors, were reviewed for a collegial decision on phenotype initially based on the 2014 proposed classification and then on the 2019 ISHLT consensus report on CLAD [17, 18]. Recipients were classified as follows: BOS, RAS, azithromycin responsive allograft dysfunction (ARAD), stable and other (death within 3 months post-transplantation, death without CLAD, insufficient data to conclude or confounding factors).

### Design of the study and recipient selection

The objective of this study was to evaluate the ability of MMP-9 blood levels to predict CLAD. We designed a comparative analysis of plasma MMP-9 concentrations at year 1 post-transplantation (Y1) between stable recipients at this time point who will develop CLAD within 2 years (i.e., by 3 years post-transplantation) and those who remained without CLAD for the same 2-year follow-up (Stable). Similarly, we designed a comparative analysis of plasma MMP-9 concentrations at year 2 post-transplantation (Y2) from stable recipients at this time point, comparing those who will develop CLAD within 2 years of sampling (i.e., by 4 years post-transplantation) and those who remained stable (Additional file 1: Figure S1).

**Fig. 1:**
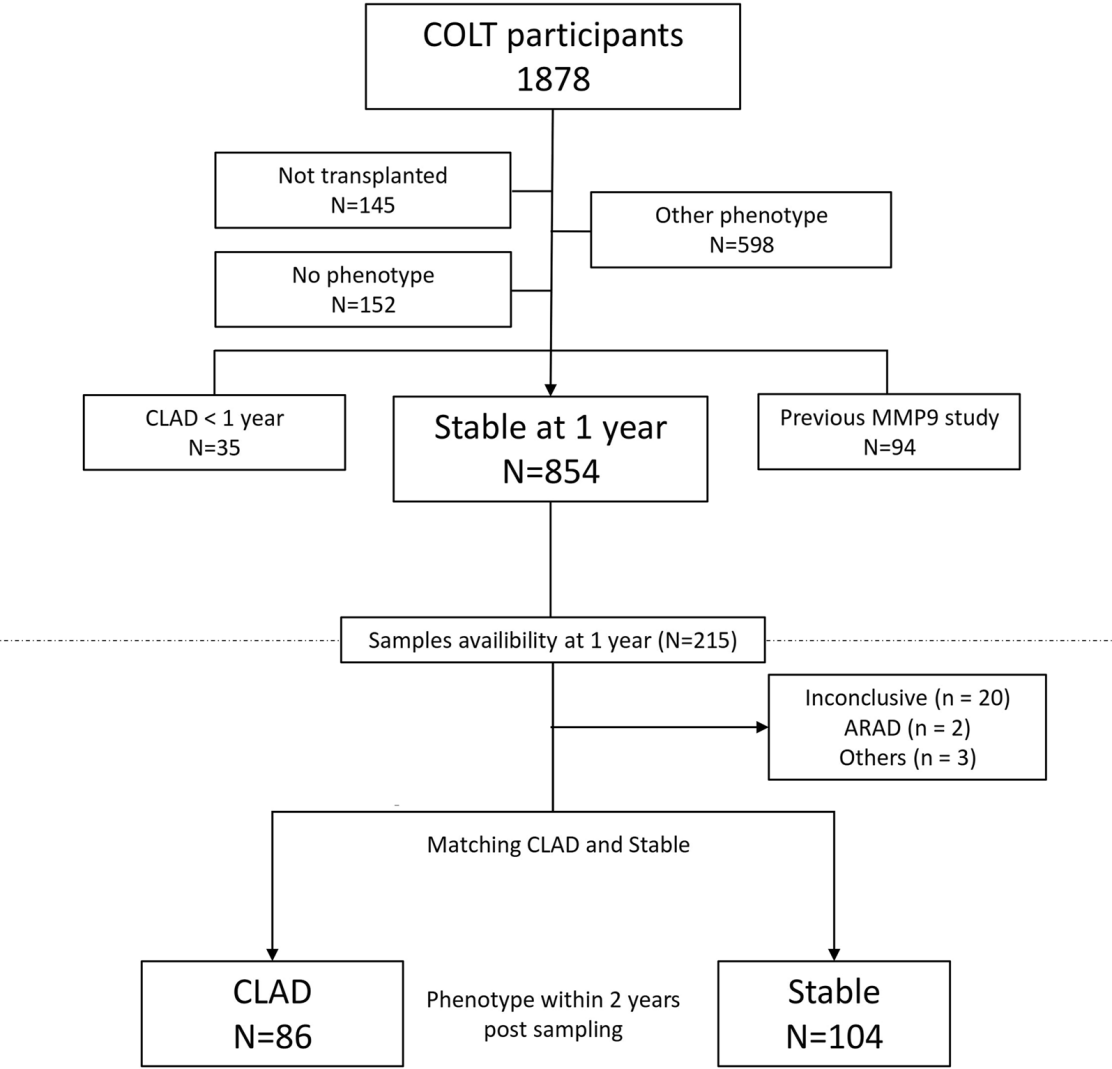
Year 1 sample analysis flowchart. Selection of COLT lung transplant recipients with CLAD or a stable phenotype. Other phenotypes included death before 3 months, death without CLAD and an inconclusive phenotype (insufficient data or cofounding factors affecting adjudication). “No phenotype” refers to recipients awaiting adjudication

**Fig. 2:**
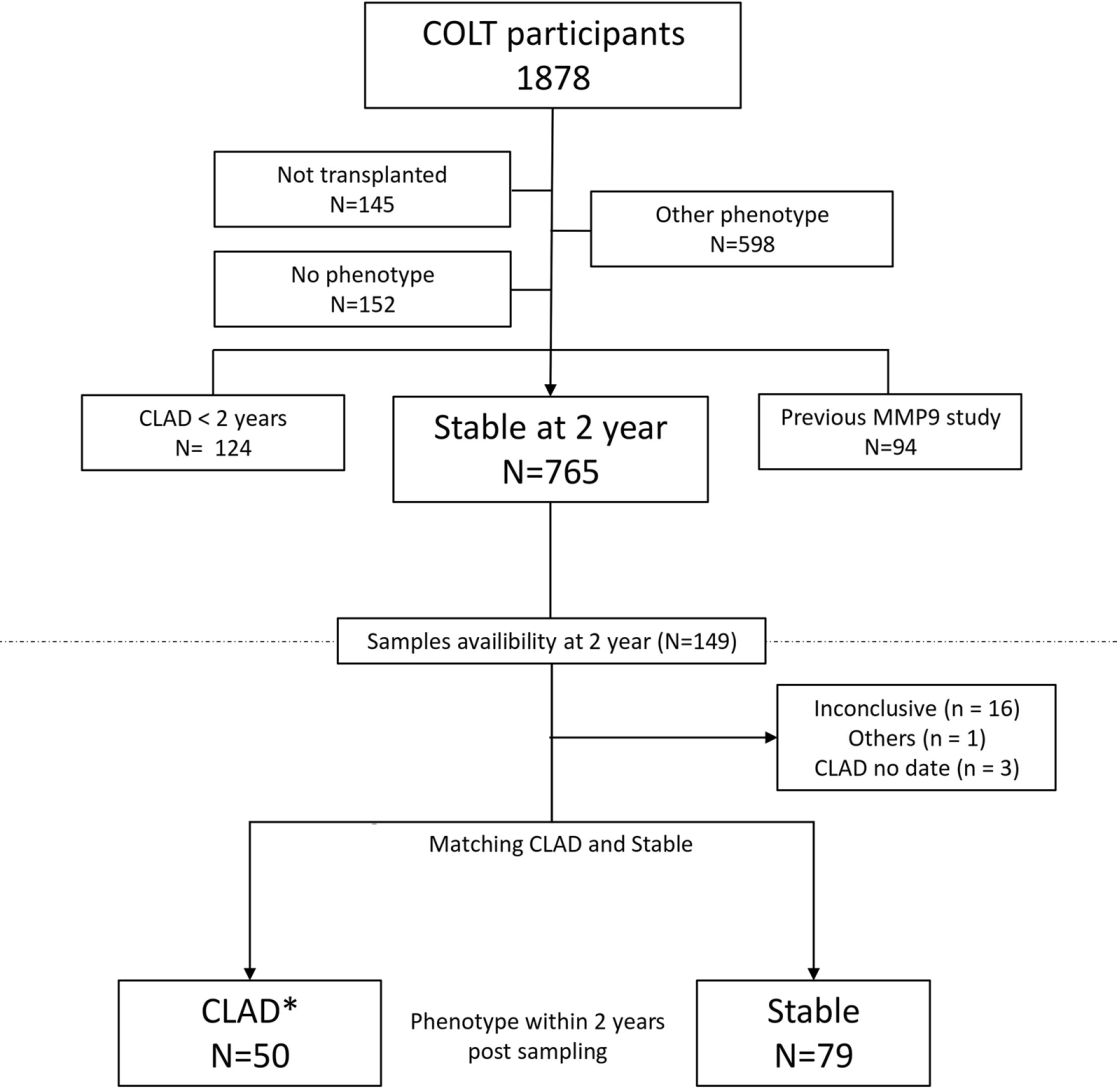
Year 2 sample flowchart. Selection of COLT lung transplant recipients with CLAD or a stable phenotype. Other phenotypes included death before 3 months, death without CLAD and an inconclusive phenotype (insufficient data or cofounding factors affecting the adjudication). “No phenotypes” refer to recipients awaiting adjudication.* One recipient was initially classified as having azithromycin-responsive allograft dysfunction and was not included in the stable group for the year 1 sample analysis but later developed CLAD and was included in the CLAD group for the year 2 sample analysis

For this purpose, we identified every COLT participant with stable lung function at Y1 for whom we had a plasma sample available, and we then selected those who presented a CLAD phenotype by year 3. Then, we matched up to 2 stable controls for each CLAD recipient on age at transplantation and gender (variable ratio matching) (Fig. 1). We excluded COLT participants who were not transplanted, recipients with the phenotype “Other” and those with no phenotype. We also excluded recipients who were included in our previous MMP-9 analysis (n = 94) [15]. According to the study design, recipients who developed CLAD before 1 year post-transplantation were not included in the Y1 analysis (n = 35), and similarly, recipients who developed CLAD before 2 years post-transplantation were not included in the Y2 analysis (n = 124). Flowcharts are represented in Fig. 1 for the Y1 analysis and Fig. 2 for the Y2 analysis. Overall, the median follow-up time from LTx was 45 months, and 63 patients were included in both the Y1 and Y2 analyses (recipients for whom a sample was available at Y1 and Y2).

### MMP-9 detection

All plasma samples were obtained in heparinized tubes, stored at − 80 °C in Nantes University Hospital Biological Resource Center and never thawed before use. MMP-9 concentrations were determined with the quantitative MMP-9 Human ELISA Kit (Thermo Fisher Scientific, BMS2016-2) according to the manufacturer’s instructions.

### Statistical analysis

For descriptive analysis, normally distributed continuous variables are summarized herein by the mean plus standard deviation, whereas nonnormally distributed continuous variables are summarized using the median and interquartile range (IQR). Categorical variables are presented as the effectiveness and percentage of modalities. We used usual statistical tests to compare distributions between two groups (Student’s t test, Mann‒Whitney-Wilcoxon test and Fisher exact test). Missing values are systematically presented. No imputation was performed.

To assess whether MMP-9 could be prognostic of CLAD, we only considered measures that had been made before the diagnosis. In addition, we drew ROC and precision-recall (PR) curves to investigate with appropriate metrics whether MMP-9 concentrations could be relevant in predicting CLAD in these patients. The confidence interval of the ROC area under the curve (AUC) and PR-AUC was computed using 3000 bootstrap iterations of the corresponding study sample. Finally, a multivariate linear regression model was fit to explain MMP-9 concentrations at Y1 by the status regarding CLAD 2 years later, plus susceptible confounders identified by a group of clinicians. Additional descriptions of the variables of interest are provided in Additional file 1: S2. We also fitted a model for MMP-9 concentrations at Y2 explained by the status regarding CLAD 2 years after, plus the same confounders. Due to a lack of data, we were unable to split our dataset into a training set and a test set. Analyses were performed using R package version 4.2.2, especially using the *tidyverse* package for data manipulation and visualization and *tidymodels* for predictive modelling.

## Results

### Prognostic value of MMP-9 at Y1

A total of 190 stable recipients were included for the analysis of MMP-9 at Y1 post-transplantation of whom 86 developed CLAD (68 BOS, 9 RAS and 9 mixed) and 104 remained stable within the next 2 years (Fig. 2). Pretransplant characteristics and early posttransplantation outcomes (within the first year post-Tx) are presented in Table 1. The distribution of underlying disease was significantly different between the stable and CLAD groups, with fewer patients with chronic obstructive pulmonary disease (COPD)/emphysema and more with interstitial lung disease (ILD) and other diagnoses in the CLAD group (p = 0.01). The median height was lower in the CLAD group (165 cm vs. 168 cm, p = 0.05), and more patients in the stable group had pre-Tx pulmonary colonization to *Candida albicans* (37% vs. 18%, p = 0.04). There were also fewer recipients in the CLAD group who benefited from induction treatment after transplantation (70% vs. 84%, p = 0.03). Finally, there was a tendency for an increased number of experienced acute cellular rejections in the CLAD group (43% vs. 30%, p = 0.08). The 2 groups were comparable in terms of sex, age, type of procedure, donor and graft characteristics and infection post-transplantation outcomes.

**Table 1:** Recipients and donor characteristics for the year-1 MMP-9 comparative analysis

	Stable (n = 104)	CLAD (n = 86)	p value	Missing value (%)
Gender male	61 (58.7)	45 (52.3)	0.5	0
Age	52 [36, 60]	51 [28, 58]	0.4	0
Height (cm)	168 (8)	165 (8)	0.05	0.5
Weight (kg)	61(15)	59 (13)	0.3	0.5
Underlying disease			0.01	0
COPD/emphysema	55 (52.9)	33 (38.4)		
Cystic fibrosis	30 (28.8)	23 (26.7)		
ILD	16 (15.4)	17 (19.8)		
Other	3 (2.9)	13 (15.1)		
Bacterial colo. pre Tx	51 (49.0)	43 (50.0)	1	0
Fungal colo. pre Tx	55 (52.9)	39 (45.3)	0.4	0
C. Albicans colo. pre Tx	37 (35.6)	18 (20.9)	0.04	0
DSA pre Tx	19 (24.7)	21 (32.8)	0.4	25.8
High emergency	8 (7.7)	6 (7.0)	1	0
Procedure			0.3	1.1
Double lung	87 (84.5)	70 (82.4)		
Heart and lung	0 (0.0)	2 (2.4)		
Single lung	16 (15.5)	13 (15.3)		
Surgical approach			0.2	4.7
Clam shell	18 (18.2)	8 (9.8)		
Sternotomy	2 (2.0)	4 (4.9)		
Thoracotomy	79 (79.8)	70 (85.4)		
ECLS	42 (42.0)	30 (35.7)	0.5	3.2
Graft cold ischemia (min)	322 [270, 380]	345 [280, 405]	0.12	3.7
Donor age	44 [30, 54]	46 [25, 57]	0.7	0.5
Donor gender male	62 (59.6)	53 (62.4)	0.8	0.5
Donor smoking habit	37 (35.9)	30 (36.6)	1	2.6
PaO2/FiO2	400 (106)	380 (107)	0.2	0.5
Mismatch CMV	16 (15.4)	16 (18.6)	0.7	0
Mismatch EBV	6 (5.8)	7 (8.1)	0.7	0
Dialysis	5 (5.1)	2 (2.4)	0.6	3.7
Induction treatment	87 (83.7)	60 (69.8)	0.03	0
Type of Induction			0.03	0
Anti-IL2	36 (34.6)	20 (23.3)		
ATG	51 (49.0)	39 (45.3)		
None	17 (16.3)	27 (31.4)		
Resp bacterial inf post Tx			0.3	0
0	19 (18.3)	21 (24.4)		
1	40 (38.5)	24 (27.9)		
2	31 (29.8)	24 (27.9)		
3	14 (13.5)	17 (19.8)		
Resp fungal inf post Tx			0.7	0
0	55 (52.9)	43 (50.0)		
1	34 (32.7)	34 (39.5)		
2	12 (11.5)	7 (8.1)		
3	3 (2.9)	2 (2.3)		
Resp viral inf post Tx			0.3	0
0	66 (63.5)	50 (58.1)		
1	28 (26.9)	25 (29.1)		
2	6 (5.8)	10 (11.6)		
3	4 (3.8)	1 (1.2)		
ACR (≥ 1)	31 (29.8)	37 (43.0)	0.08	0
AMR	3 (2.9)	7 (8.1)	0.2	0

Results are expressed in median with interquartile range, mean with standard deviation or n and %. *CLAD* chronic lung allograft dysfunction, COPD: chronic obstructive pulmonary disease. ILD: interstitial lung disease. Other: other underlying diagnosis (pulmonary hypertension, sarcoidosis, connective tissue disease, bronchiectasis). Bacterial colo. pre Tx: bacterial colonization pre transplantation. Fungal colo. pre Tx. Fungal colonization pre transplantation. DSA: donor specific antibody. ECLS: extracorporeal life support per transplantation. CMV: cytomegalovirus. EBV: Epstein-Barr virus. ATG: rabbit antithymoglobulin. Anti-IL2: anti interleukine-2. Resp bacterial inf post Tx: lower respiratory tract bacterial infection in the first year post transplantation, number of episode. Resp fungal inf post Tx: lower respiratory tract fungal infection in the first year post transplantation, number of episode. ACR: acute cellular rejection, number of patients with one or more episode in the first year post transplantation. AMR: antibody mediated rejection, number of patients with one or more episode in the first year post transplantation

**Fig. 3:**
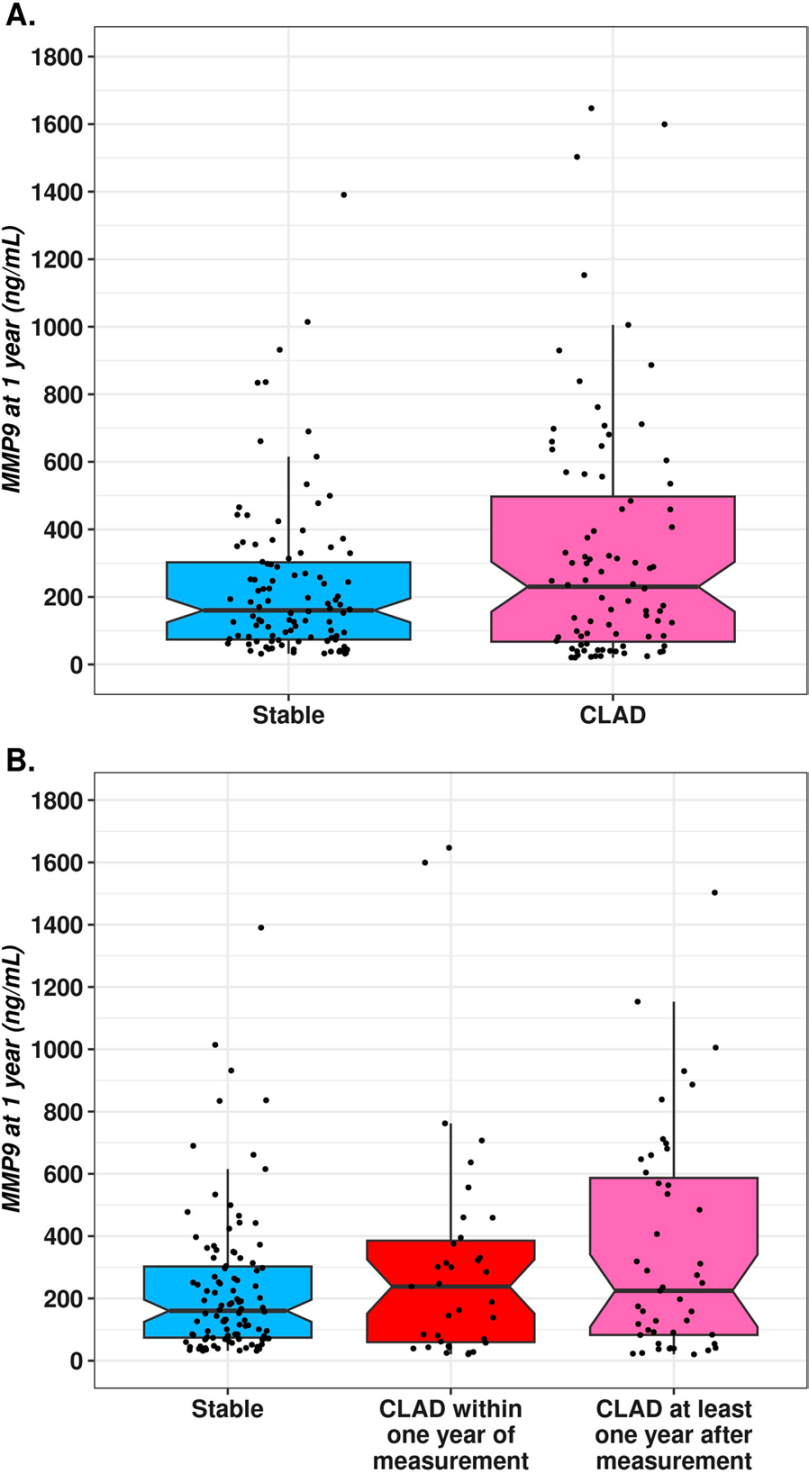
Blood MMP-9 value at Y1. **A** Comparison of the MMP-9 plasmatic concentration at Y1 between recipients who then developed CLAD and those who remained stable in the next 2 years (p = 0.2). **B** Comparison of samples close to CLAD and distant from CLAD. Comparison of the MMP-9 plasmatic concentration at Y1 between recipients who developed CLAD within 1 year of measurement, those who developed CLAD at least 1 year after measurement and those who remained stable at least 2 years after measurement. No significant difference was observed. Boxplot interpretation: thick horizontal bars correspond to the median, notches to the upper and lower limits of the confidence interval and top and bottom of the box to the first and third interquartile of MMP-9 values

Before transplantation, the MMP-9 levels were statistically similar in both the CLAD and stable groups (386 ng/ml _IQR_[174–757] vs. 349 ng/m _IQR_[162–822], p = 0.8). The MMP-9 plasma median concentration at Y1 was 237 ng/ml _IQR_[72–551] for recipients who developed CLAD within the next 2 years and 165 ng/ml _IQR_[75–317] for those who remained stable (Fig. 3). The difference was not significant (p = 0.2). Additionally, there was no significant difference according to CLAD phenotype (Additional file 1: Figure S2). In this analysis, we also distinguished those who developed CLAD within the year that followed the Y1 measurement (sample close to CLAD) and those who developed CLAD at least 1 year later (sample distant from CLAD). In this setting, the MMP-9 blood concentration was slightly higher when the sample was close to CLAD than when the sample was distant from CLAD diagnosis (248 ng/ml vs. 225 ng/ml, p = 0.9) (Fig. 3). The multivariate analysis revealed that the CLAD phenotype was associated with an increase in MMP-9 plasma levels, but the difference did not reach statistical significance (average increase of 126, 95% CI [− 32–284], p = 0.1) (Table 2). To test the discriminating capacity of Y2 MMP-9 blood measurement for CLAD prediction, we performed a PR curve, which showed an AUC of 53% (95% CI [43–65]). For the ROC curve, we found an AUC of 55% (95% CI [47–63]) (Additional file 1: Figure S3).

**Table 2:** Multivariate analysis of factors influencing MMP-9 blood levels at Y1 and Y2

Predictors	Y1 analysis			Y2 analysis		
	MMP-9 estimates	CI	p	MMP-9 estimates	CI	p
CLAD (vs Stable)	123.32	− 34–281	0.1	150.9	20–281	0.02
Cystic Fibrosis	1.08	− 193–195	1	− 112.51	− 254–29	0.1
ILD	− 39.11	− 247–169	0.7	21.73	− 149–192	0.8
Other	− 18.32	− 301–264	0.9	− 186.44	− 481–108	0.2
CMV mismatch	− 56.47	− 260–147	0.6	− 36.83	− 191–117	0.6
Fungal colo. pre Tx	− 12.69	− 163–138	0.9	129.13	1.7–257	0.05
Graft cold ischemia	− 0.51	− 1.3–0.3	0.2	− 0.46	− 1.2–0.3	0.2
Donor PaO2/FiO2	− 0.53	− 1.2–0.2	0.1	0.2	− 0.4–0.8	0.5
Induction treatment	− 61.32	− 241–118	0.5	− 108.01	− 260–44	0.1
ACR (> 1)	− 98.6	− 257–60	0.2	40.23	− 92–173	0.5
Bacterial infection (> 1)	− 6.23	− 197–184	0.9	97.94	− 59–255	0.2
Fungal infection (> 1)	147.54	− 10–305	0.07	− 28.73	− 156–98	0.6
Viral infection (> 1)	46.46	− 106–199	0.5	− 60.8	− 187–66	0.3

CLAD: chronic lung allograft dysfunction. ILD: interstitial lung disease. Other: other underlying diagnosis (pulmonary hypertension, sarcoidosis, connective tissue disease, bronchiectasis). The reference chose for underlying disease was COPD/emphysema. CMV: cytomegalovirus, mismatch referring to a transplantation with a recipient with negative serology and a donor with positive serology to CMV. ACR: acute cellular rejection, one or more episode within the first year post transplantation. The infection factors refers to bacterial, fungal or viral infection of the lower respiratory tract, one or more episode within the first year post transplantation

### Prognostic value of MMP-9 at Y2

For this analysis, 129 LTx recipients were included, of whom 50 developed CLAD and 79 remained stable within the next 2 years (i.e., up to 4 years post-transplantation) (Fig. 2). Notably, one patient was initially diagnosed with ARAD (and thus not included in the stable group) but later developed CLAD and was included in the CLAD Y2 group. Pretransplantation characteristics, per transplantation data and early post-transplantation outcomes are presented in Table 3. Few differences can be noted compared to the Y1 analysis. Underlying diseases were not differently distributed between the CLAD and stable groups, and there was no difference in recipient height. Interestingly, there were more CLAD patients with pre-Tx colonization with *Pseudomonas aeruginosa* (46% vs. 27%, p = 0.04) and fewer with *Candida albicans* (16% vs. 37%, p = 0.02). The 2 groups were comparable in terms of sex, age, type of procedure, donor and graft characteristics, infection and acute rejection outcomes post-transplantation.

**Table 3:** Recipients and donor characteristics for the year-2 MMP-9 comparative analysis

	Stable (n = 79)	CLAD (n = 50)	p	Missing (%)
Gender male	49 (62.0)	32 (64.0)	1	0
Age	49 [31, 59]	46.50 [27.25, 58.00]	0.4	0
Height (cm)	167.23 (7.44)	168.70 (9.09)	0.3	0
Weight (kg)	58.37 (14.27)	58.86 (13.64)	0.8	0
Underlying disease			0.06	0
COPD/emphysema	34 (43.0)	17 (34.0)		
Cystic fibrosis	28 (35.4)	19 (38.0)		
ILD	16 (20.3)	8 (16.0)		
Other	1 (1.3)	6 (12.0)		
Bacterial colo. pre Tx	42 (53.2)	28 (56.0)	0.9	0
*S. Aureus* colo. pre Tx	13 (16.5)	7 (14.0)	0.9	0
*P. Aeruginosa* colo. pre Tx	21 (27)	23 (46)	0.04	0
Fungal colo. pre Tx	40 (50.6)	22 (44.0)	0.6	0
*A. fumigatu*s colo. pre Tx	14 (17.7)	11 (22.0)	0.7	0
*C. Albicans* colo. pre Tx	29 (37)	8 (16)	0.02	0
DSA pre TX	17 (27.9)	15 (36.6)	0.5	20.9
High emergency	9 (11.4)	2 (4.0)	0.2	0
Procedure	12 (15.6)	8 (16.3)	1	2.3
Double lung				
Heart and lung				
Single lung				
Surgical approach			0.6	1.6
Clam shell	15 (19.2)	8 (16.3)		
Sternotomy	1 (1.3)	2 (4.1)		
Thoracotomy	62 (79.5)	39 (79.6)		
ECLS	36 (46.8)	22 (44.0)	0.9	1.6
Cold ischemia (min)	330 [275, 380]	340 [260, 395]	0.8	2.3
Donor age	44 [26, 53]	46 [36, 57]	0.3	0
Donor gender male	45 (57)	33 (66)	0.4	0
Donor smoking habit	28 (35.4)	19 (38.8)	0.8	0.8
PaO_2_/FiO_2_	393.27 (104.85)	392.46 (96.27)	1	1.6
Mismatch CMV	17 (21.5)	11 (22.0)	1	0
Mismatch EBV	6 (7.6)	8 (16.0)	0.2	0
Dialysis	4 (5.3)	3 (6.1)	1	3.9
Induction treatment	63 (79.7)	37 (74.0)	0.6	0
Type of induction			0.4	0
Anti-IL2	28 (35.4)	12 (24.0)		
ATG	35 (44.3)	25 (50.0)		
None	16 (20.3)	13 (26.0)		
Resp bacterial inf post Tx			0.1	0
0	13 (16.5)	11 (22.0)		
1	32 (40.5)	14 (28.0)		
2	24 (30.4)	12 (24.0)		
3	10 (12.7)	13 (26.0)		
Resp fungal inf post Tx			0.3	0
0	46 (58.2)	21 (42.0)		
1	23 (29.1)	19 (38.0)		
2	8 (10.1)	7 (14.0)		
3	2 (2.5)	3 (6.0)		
Resp viral inf post Tx			0.1	0
0	55 (69.6)	25 (50.0)		
1	18 (22.8)	19 (38.0)		
2	4 (5.1)	5 (10.0)		
3	2 (2.5)	1 (2.0)		
ACR (≥ 1)	24 (30.4)	22 (44.0)	0.2	0
AMR	4 (5.1)	3 (6.0)	1	0

Results are expressed in median with interquartile range, mean with standard deviation or n and %. CLAD: chronic lung allograft dysfunction. COPD: chronic obstructive pulmonary disease. ILD: interstitial lung disease. Other: other underlying diagnosis (pulmonary hypertension, sarcoidosis, connective tissue disease, bronchiectasis). Bacterial colo. pre Tx: bacterial colonization pre transplantation. Fungal colo. pre Tx. Fungal colonization pre transplantation. DSA: donor specific antibody. ECLS: extracorporeal life support per transplantation. CMV: cytomegalovirus. EBV: Epstein-Barr virus. ATG: rabbit antithymoglobulin. Anti-IL2: anti interleukine-2. Resp bacterial inf post Tx: lower respiratory tract bacterial infection in the first year post transplantation, number of episode. Resp fungal inf post Tx: lower respiratory tract fungal infection in the first year post transplantation, number of episode. ACR: acute cellular rejection, number of patients with one or more episode in the first year post transplantation. AMR: antibody mediated rejection, number of patients with one or more episode in the first year post transplantation

**Fig. 4:**
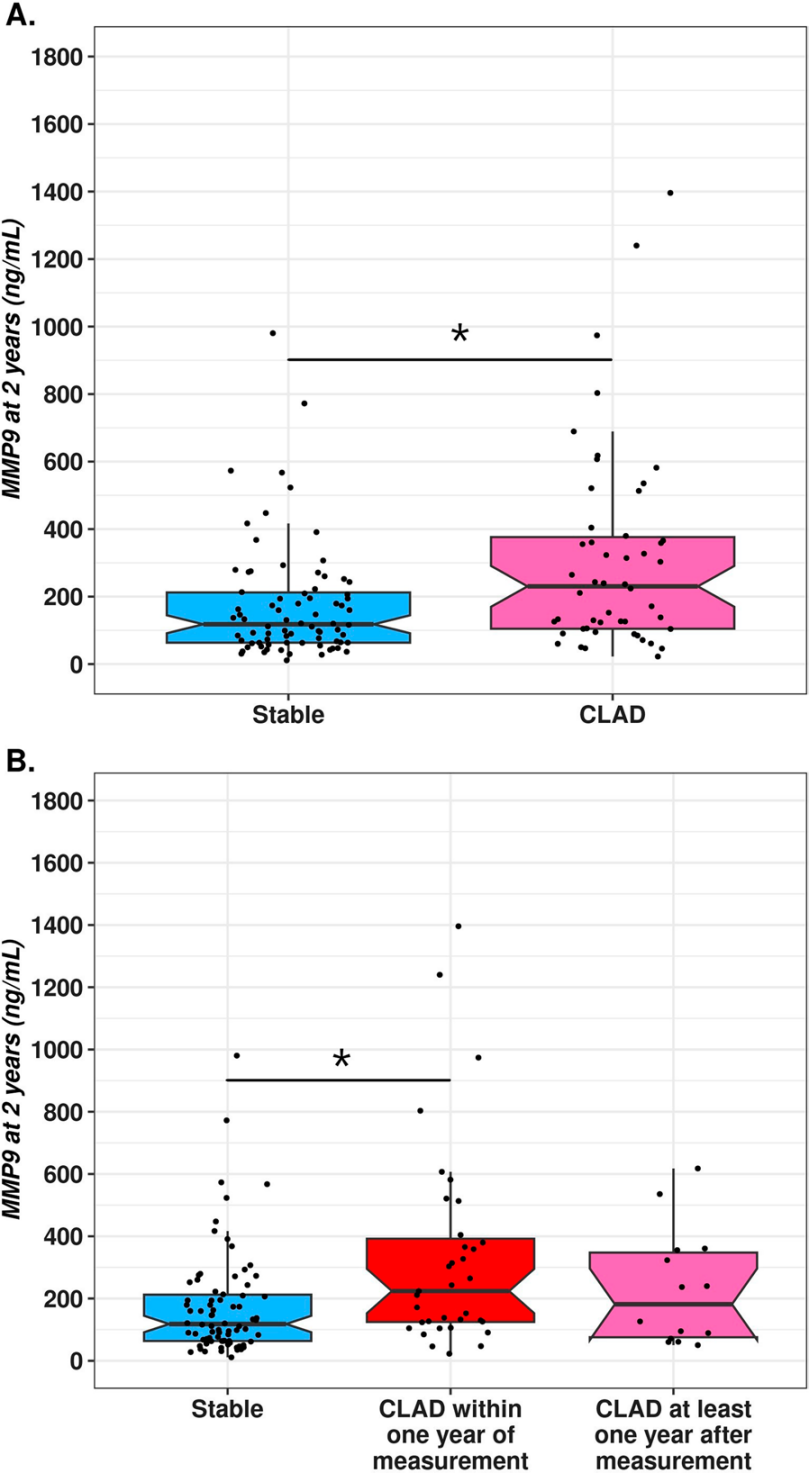
Blood MMP-9 value at Y2. **A** Comparison of the MMP-9 plasmatic concentration at Y2 between recipients who then developed CLAD and those who remained stable in the next 2 years (p = 0.003). **B** Blood MMP-9 value at Y2 with comparison of samples close to the CLAD and distant from the CLAD. Comparison of the MMP-9 plasmatic concentration at Y2 between recipients who developed CLAD within 1 year of measurement, those who developed CLAD at least 1 year after measurement and those who remained stable at least 2 years after measurement with a significant difference between the stable and CLAD groups within 1 year (p = 0.003). Boxplot interpretation: thick horizontal bars correspond to the median, notches to the upper and lower limits of the confidence interval and top and bottom of the box to the first and third interquartile of MMP-9 values. * corresponds to p value < 0.05

**Fig. 5:**
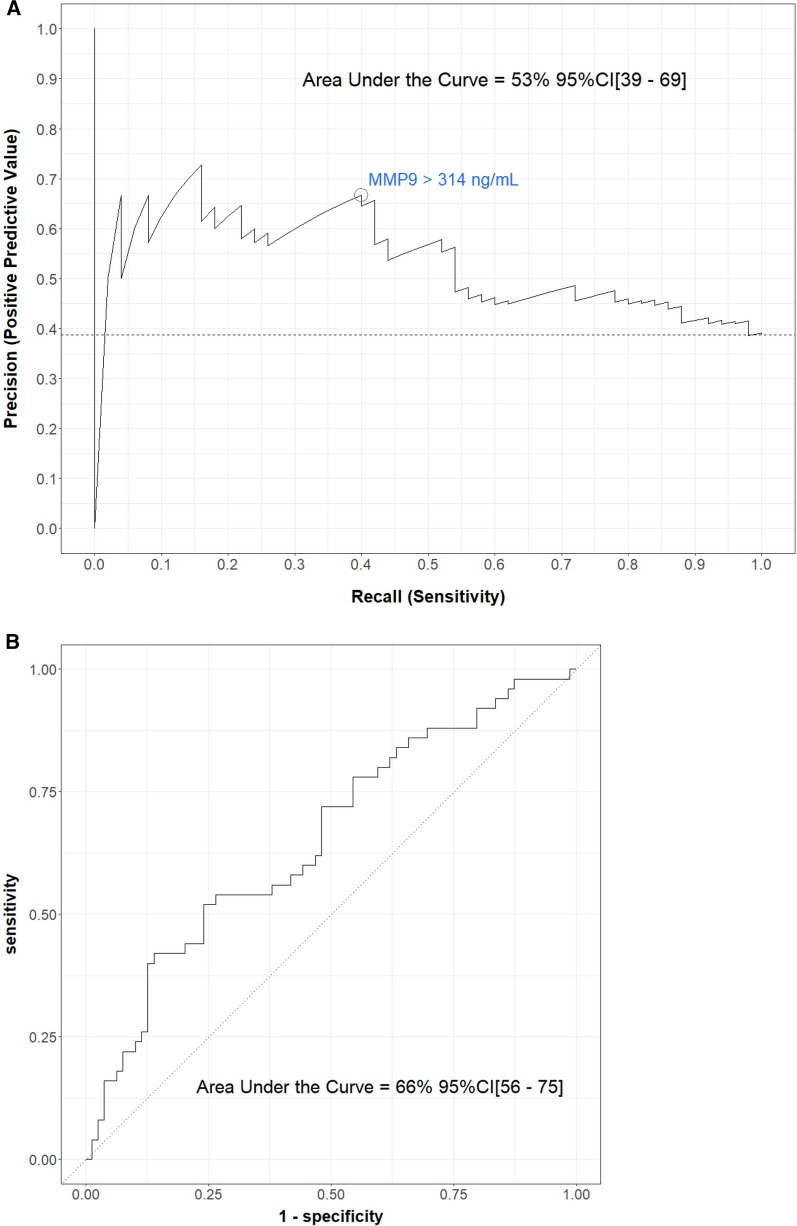
Precision-Recall (**A**) and ROC (**B**) curves for the Y1 MMP-9 analysis. The precision-recall curve represents, for each available value of MMP-9, the positive predictive value and sensitivity for CLAD onset within the next 2 years

Before transplantation, the MMP-9 levels were 419 ng/ml _IQR_[242–675] in the CLAD group and 345 ng/ml _IQR_[162–861] in the stable group (p = 0.6). Interestingly, the MMP-9 plasma median concentration at Y2 was significantly higher for recipients who developed CLAD within the next 2 years than for those who remained stable (230 ng/ml _IQR_[105–376] vs. 118 ng/ml _IQR_[64–218], p = 0.003) (Fig. 4). BOS, RAS and mixed MMP-9 values were all above the stable group, but the difference was significant only for recipients with RAS (265 ng/ml vs. 119 ng/ml, p = 0.02) (Additional file 1: Figure S4). In the CLAD group, patients with a sample close to the CLAD diagnostic date (within 1 year of measurement) had a higher blood MMP-9 level than those with a sample distant from the CLAD (at least 1 year after measurement) (224 ng/ml vs. 182 ng/ml, Fig. 4). In the multivariate analysis, higher MMP-9 levels at Y2 were independently and significantly associated with future CLAD diagnosis (estimated increase of 151 ng/ml, 95% CI [20–281], p = 0.02) (Table 2). The only other factor for which a significant difference was observed was pretransplantation colonization with *Candida albicans,* which resulted in an average increase in MMP-9 concentration at Y1 of 129 ng/mL (95% CI [2–257, (p = 0.04) (Table 3). To test the discriminating capacity of Y2 MMP-9 blood measurement for CLAD prediction, we performed a PR curve, which showed an AUC of 53% (95% CI [43–65]). For the ROC curve, we found an AUC of 66% (95% CI [56–75]) (Fig. 5). The prognostic power of blood MMP-9 was demonstrated at higher values. For example, an MMP-9 blood level higher than 314 ng/ml in our cohort enabled the identification of 40% of CLAD cases (recall or sensitivity) with a precision (positive predictive value) of 65%. Finally, to determine the prognostic effect of plasma MMP9 kinetics, we calculated the individual difference between MMP9 at Y1 and MMP9 at Y2 (MMP9 Y1–MMP9 Y2). We had 63 recipients in the CLAD group and 32 recipients in the stable group for which we had a sample available at Y1 and Y2. Interestingly, we observed an average individual decrease of 100 ng/ml in the stable group, whereas it remained almost similar in the CLAD group, with a calculated difference of 1 ng/ml. The difference between the two groups was, however, not significant (p = 0.9). The longitudinal analysis of MMP9 levels before transplantation, at Y1 and Y2 for participants with available samples at those 3 time points is presented in Additional file 1: Figure S5.

## Discussion

In this study, we report a potential prognostic value of MMP-9 plasma concentrations at 2 years post-transplantation for CLAD diagnosis. In a smaller set of patients, our team, with a different methodology in which the reference time point was CLAD diagnosis, found increased levels of plasma MMP-9 1 year before CLAD diagnosis compared to the levels in recipients without CLAD [15]. It was, however, essential to validate the prognostic potential of blood MMP-9 in a setting that could enable risk stratification and thus therapeutic intervention. To this aim, we chose two posttransplantation time points, Y1 and Y2, that have clinical relevance because they are sufficiently distant from the lung transplantation surgery, early infection and immune complications that could strongly interfere with inflammatory or wound-healing-related biomarkers but also early enough regarding the CLAD onset time to allow early intervention in the lung transplant population.

The reason MMP-9 levels predict Y2 but not Y1 CLAD risk is unclear. However, it could be related to the fact that MMP-9 plasma levels seem to be higher when CLAD appears within 1 year from the date of sampling because we also observed that the average time between the sample and CLAD diagnosis was longer in the Y1 analysis than in the Y2 analysis (643 days vs. 489 days, respectively). The Louven group indeed described a median time of CLAD onset of 3.2 years post-transplantation, and in a North American population, Kulkarni et al. reported a median BOS-free survival of 3.6 years for double lung transplant recipients [19, 20]. The only study that prospectively evaluated MMP-9 levels after transplantation was performed by Kastelijn et al. on 10 LTx recipients who developed BOS and 10 matched control recipients without BOS. They found that the median MMP-9 concentrations in all serial samples were significantly higher in BOS patients [190 ng/ml (163–238) versus 128 ng/ml (106–162), p < 0.0001], but a longitudinal analysis of MMP-9 serum levels from the time of transplantation onwards did not reveal a significant difference in serum levels in the period preceding BOS [21]. More recently, a study using a high-component multiplex immunoassay identified MMP-9, among other proteins, as a diagnostic biomarker of BOS in a set of patients (n = 46). This finding was confirmed in another set of patients from different centres by ELISA [8]. In the different context of BOS related to chronic graft­versus-host disease after allogeneic haematopoietic cell transplantation, plasma MMP-9 was also found at a higher concentration in 33 patients with BOS at the time of diagnosis than in 60 recipients without BOS, and elevated MMP-9 was thought to be associated with treatment failure and worse prognosis [22].

In the literature, more data are available for MMP-9 in BAL. In a cohort of 45 recipients, Vandemeulen et al. found that MMP9 levels were increased in RAS and BOS at the time of diagnosis [23]. Similarly, Heijink et al. found increased levels of MMP-2, MMP-3, MMP-7, MMP-8, MMP-9 and the MMP endogenous inhibitor TIMP1 at the time of diagnosis [11]. Older works have shown the predictive potential of MMP-9 in BAL in a relatively smaller cohort of less than 25 patients and not at a prespecified time point that could apply in real-life surveillance [12, 13].

Thus, regarding all these data, we are the first to describe blood MMP-9 levels in a large cohort of LTx recipients as prognostic of CLAD onset with a 66% AUC in the ROC analysis and 53% in the PR analysis. These figures may appear as low, likely due to the high variability of MMP-9 blood levels within each group of patients. While remaining a limitation, if considered alone for a given patient, no parameter currently used can actually enable a prognostic assessment of CLAD. Additionally, one important point is that given the time points chosen, i.e., 1 and 2 years for MMP-9 measurement, early CLAD (before 1-year post-transplantation) were not in the spectrum of this study. To answer these limitations, first, we did not consider MMP-9 alone as a predictive biomarker of CLAD, but this work clearly demonstrates its potential to increase the efficiency of a CLAD multidimensional score. Our group previously reported a blood gene expression analysis in which we identified three genes, POU class 2 associating factor 1, T-cell leukaemia/lymphoma protein 1A and B-cell lymphocyte kinase, which were validated as predictive biomarkers of BOS more than 6 months before diagnosis [24]. We also found differential T and B lymphocyte phenotyping between CLAD and stable recipients, again upstream of CLAD diagnosis [25, 26]. Analysis of those potential biomarkers with blood MMP-9 at this specific Y2 time point in particular will be of interest in the ongoing new prospective multicentric study to build a multidimensional risk stratification for CLAD, along with demographic, clinical and environmental data (PRELUD study, NCT03967340).

## Conclusion

We described here in a large cohort the interesting prognostic potential of blood MMP-9 levels measured at 1 and 2 years post-transplantation. The implementation of these mini-invasive biomarkers into a multidimensional score, along with transcriptomic and immune phenotyping, has the potential to provide an efficient risk stratification tool to patients and clinicians.

### Supplementary Information

**Additional file 1: S1.** COLT study protocol. **S2.** Description of variables of interest. **Figure S1.** Study protocol. **Figure S2.** Comparison of MMP-9 blood levels of Y2 analysis according to CLAD phenotypes. **Figure S3.** Precision-Recall (A) and ROC (B) curves for the Y1 MMP-9 analysis. **Figure S4.** Comparison of MMP-9 blood levels of Y2 analysis according to CLAD phenotypes. **Figure S5.** Longitudinal analysis of MMP-9 blood levels for recipients with available samples before transplantation, at Y1 and Y2.

## Data Availability

Any datasets used can be accessed after request to the corresponding author via e-mail.

## Abbreviations

ARAD: Azithromycin-responsive allograft dysfunction
AUC: Area under the curve
BAL: Bronchoalveolar lavage
BOS: Bronchiolitis obliterans syndrome
CLAD: Chronic lung allograft dysfunction
COLT: Cohort in lung transplantation
COPD: Chronic obstructive pulmonary disease
ILD: Interstitial lung disease
LTx: Lung transplantation
MMP-9: Matrix metalloproteinase 9
PR: Precision-recall
RAS: Restrictive allograft syndrome
