# Supplementary material for: Blood MMP-9 measured at 2 years after lung transplantation as a prognostic biomarker of chronic lung allograft dysfunction
Source: Respir Res. 2024 Feb 9;25:88. doi: 10.1186/s12931-024-02707-3 (PMC10858575; doi:10.1186/s12931-024-02707-3)

**Blood MMP-9 measured at two-year post lung transplantation as a prognostic biomarker of chronic lung allograft dysfunction.**

Additional Appendix Material

[Additional S1. COLT study protocol 2](#_Toc155797355)

[Additional S2. Description of variables of interest 7](#_Toc155797356)

[Figure S1. Study protocol 8](#_Toc155797357)

[Figure S2. Comparison of MMP-9 blood levels of Y2 analysis according to CLAD phenotypes. 9](#_Toc155797358)

[Figure S3. Precision-Recall (A) and ROC (B) curves for the Y1 MMP-9 analysis 10](#_Toc155797359)

[Figure S4. Comparison of MMP-9 blood levels of Y2 analysis according to CLAD phenotypes. 11](#_Toc155797360)

[Figure S5. Longitudinal analysis of MMP-9 blood levels for recipients with available samples before transplantation, at Y1 and Y2. 12](#_Toc155797361)

# Additional S1. COLT study protocol

**Study Objective**

The goal of the COLT study is to investigate predictive factors for chronic lung allograft dysfunction. It relies on two cornerstones: to share between centers relevant clinical, biological and functional data and to create a prospective biocollection. The main objective is to identify biomarkers of CLAD. Secondary objectives are to investigate the precise physiopathology of CLAD and to enable studies on lung transplantation on other theme (cancer or infectious disease related to transplantation)

**Methodology**

Study Design

COLT is a prospective multicentric cohort study of CLAD development in patients followed-up 10 years post transplantation. All French lung transplantation team participate plus the Belgium centre of Brussels. Nantes is the coordinator centre and Pr Antoine Magnan is the principal investigator. The COLT study involves the pooling by all participant centres of clinical, functional, radiological and biological data in a prospective, homogenous and standardized manner in a dedicated shared database. It is associated with a prospectively set up biocollection including plasma, serum, tissu, bronchio-alveolar lavage (BAL), RNA and DNA samples. The study protocol involves planned follow-up visit in which clinical, biological and functional data are implemented in the electronic case report form and blood samples are taken from patient. The protocol does not interfere further with the usual centre’s medical care.

This research was conducted in accordance with the Declaration of Helsinki and complies with:

- Articles L. 1121-1 to L. 1126-12 of the French Public Health Code concerning research involving the human body.

- the French Data Protection Act of January 6, 1978, as amended, and Act no. 2018-493 of June 20, 2018 on the protection of personal data

- Regulation (EU) 2016/679 of the European Parliament and of the Council of 27 April 2016 on the protection of individuals with regard to the processing of personal data and on the free movement of such data (RGPD).

The protocol has received the approval of the Ethic Committee on 24th February 2009, and the authorization from the French regulatory authority (AFSSASP/ANSM) on 5th February 2009. The protocol is also registered to the CNIL (national committee for data processing and liberties) and approved on 16th September 2011. The University Hospital Ethical Committee of Nantes and the Committee for the Protection of Patients from Biologic Risks approved this study.

Study population

The study aims to include every patient eligible for lung transplantation in any of the adult lun g transplantation centres of Hôpital Foch (Suresnes), Marseille, Strasbourg, Centre Chirurgical Marie Lannelongue (Le Plessis Robinson), Nantes, Hôpital Bichat (Paris), Hôpital Européen Georges Pompidou (Paris), Bordeaux, Lyon, Toulouse, Grenoble and Brussels. Information on data collection and final use of samples are given and the written consent obtained during the pre-transplantation assessment and patients are included at the time of listing, *i.e.* before transplantation. Patients without a social insurance, unable to follow the protocol, to give an informed consent for the biocollection, nursing or pregnant women or patient with a concomitant inflammatory disease were not included. In the specific situation of the high-emergency list, the consent is gathered from the patient’s trust person or the closest next of kin after information on the study and the patient’s consent is obtain after transplantation. In case of refusal to carry on the study, the patient is withdrew and all samples associated are destroyed.

Data collection

At inclusion (V0) a physical examination and pulmonary function test are performed. Demographic, biological and clinical data are compiled. The first visit (V1) is at the time of transplantation, specific information on the recipient, the donor, the surgery and immediate post-operative outcomes are implemented. The time of the following visits depends on which group the patient belongs to. There are three sets of patients: A) from the 1^st^ to the 500^th^ transplanted patients were included in the biomedical research with visits every 6 months with sample collection, additional blood samples were done for the PBMC B) from the 501^st^ to 1200^th^ patient were included in the non-interventional research with visit every year with sample collection C) from the 1201^st^ transplanted patient, follow-up every year without sample collection. Each follow-up visit gathered clinical, biological and functional information with specific items on allograft dysfunction (acute cellular rejection, chronic lung allograft dysfunction and antibody mediated rejection), complications (bronchial anastomosis, infection, renal dysfunction and tumor) and maintenance immunosuppression regimen (with mention of azithromycin) (Table 1)

CLAD adjudication committee

When the database was initially build in 2008, the CLAD entity was not yet described (verleden). In 2013, to enable a homogenous classification of our patients, we organized the first adjudication committee to ascertain their phenotype at 3 years post transplantation or before 3 years if they had died or developed CLAD. Those meeting were held face-to-face or by means of videoconference. At least seven centres were represented for each committee. Decision was based on PFTs, radiological findings (chest computed tomography scan for most, standard chest x-ray if not available), information extracted from the database for confounding factors and diagnosis according to the centre providing care to the specific patient. Phenotypes were defined as follow:

- BOS: persistent decline of FEV1 > 20% from baseline with FEV1/FVC < 0,70
- RAS: persistent decline of FEV1 > 20% from baseline with decline of TLC > 10% (or FVC > 20% if not available) and radiological fibrotic changes
- Mixed: RAS phenotype with an obstructive component or BOS phenotype with clear pulmonary fibrotic infiltrates
- Stable: FEV1 at the time of adjudication > 90% of baseline without any life-threatening condition (*e.g.* cancer)
- Inconclusive: possible CLAD or stable but with major confounding factor (concurrent cancer, severe bronchial stenosis) or insufficient data
- Other: death before 3 months post transplantation or from another cause without evidence for CLAD

Other subtypes were identified regarding the azithromycin status: the well-defined azithromycin-responsive allograft dysfunction and patient with stable lung function on azithromycin (treatment started from transplantation or without significant identified decline of FEV1). Baseline value is the mean of the two best FEV1 value performed at least 3 weeks apart. The first committee assessing patient at five years post lung transplantation was held in November 2017.

Sample collection

The biocollection concerns the first 1200 transplanted patients of the cohort (group A and B). The schedule is identical for V0 and V1, then from V2 patient from group A have a follow-up every 6 month and for group B every year. Collection of samples ends when the patient reaches 5 years post-transplant. Patient form group A, have additional blood samples (for the PBMC), 3 Li-Hep tubes of 9 ml were necessary.

At inclusion, a blood sample of 41ml is collected from the patient. Specific analysis of lymphocyte population, transcriptome and genome are provided for. Distribution is as follow:

- 2 EDTA tubes of 5 ml
- 2 Li-Hep tubes of 9 ml
- 1 Paxgene® of 3 ml
- 1 dry tube of 10 ml

At the time of transplantation, a lung biopsy of the explanted lung is performed for transcriptomic analysis. A 31 ml blood sample is gathered from the recipient just before transplantation (same distribution as in V0 without the 2 EDTA tubes). Blood sample is also retrieve from the donor with family agreement with planned lymphocyte population study. Distribution is as follow:

- 1 EDTA tubes of 4 ml
- 1 Paxgene® of 3 ml
- 1 dry tube of 9 ml

From the donor, tracheal ring can be retrieved for immediate airway epithelial cells dissociation and further primary culture. Those are carried out as needed depending on specific associated studies.

For the following visits and up to 5 years post-transplantation, blood sample is collected as in V1. BAL, transbronchial biopsies, induced sputum and exhaled breath condensate are collected as per centre protocol according to their own follow-up schedules. Some centres will perfomed systematic bronchoscopy with sampling of BAL and transbronchial biopsies as some will do it only if lung function alteration.

All samples are stored at -80°C by the Biological Resources Centre of Nantes University Hospital.

# Additional S2. Description of variables of interest

Age: age of recipients in years at the time of transplantation (similar for height and weight)

Other underlying disease: group I pulmonary hypertension, pulmonary disease related to connective tissue disease (including sclerodermia and rheumatoid arthritis), sarcoidosis, lymphagioleiomyomatosis, Langherans histiocytosis, non-cystic fibrosis bronchiectasis.

Bacterial colonization pre transplantation included colonization to pseudomonas aeruginosa, staphylococcus aureus, stenotrophomonas maltophilia, achromobacter xylosoxydans and haemophilus influenza.

Fungal colonization pretransplantation included candida ablicans and other candida species, aspergillus fumigatus, niger, terreus and scedosporium

High emergency: correspond to candidates listed on the french national priority list (also known as the “high emergency list”). Only candidates with interstitial lung disease, cystic fibrosis and pulmonary hypertension are eligible if they present specific criteria of severity (*e.g.* for candidates with cystic fibrosis, PaO2 of > 55 mmHg despite over 18h/day of non-invasive ventilation) associated with short high risk of death. The candidates listed are prioritized at the national level for 16 days.

Dialysis corresponds to recipients who required dialysis immediately after transplantation, before the first discharge from hospital.

Induction treatment: medical treatment first administrated during transplantation procedure to avoid hyperacute allograft rejection and immediate post-operative acute cellular rejection. The only two drugs used in this study were the monoclonal anti-IL-2 antibody Basiliximab and rabbit anti-thymoglobuline.

# Figure S1. Study protocol


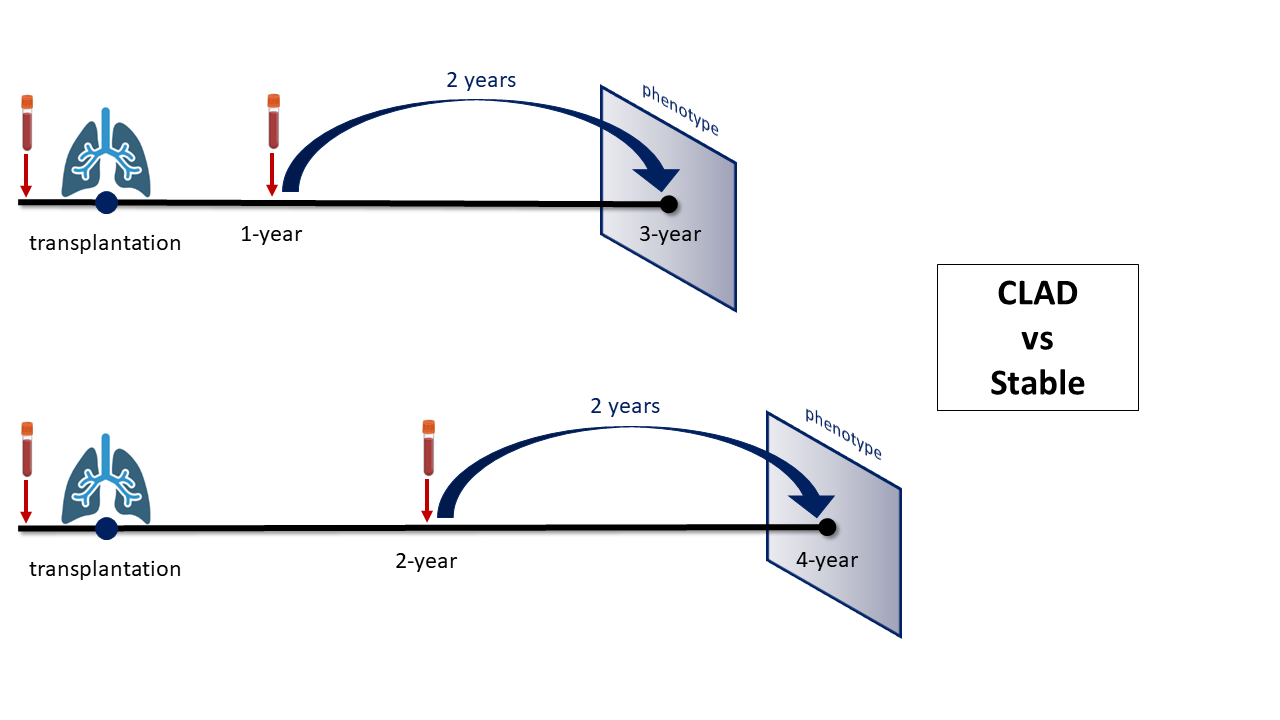


Recipients had blood taken at 1-year and/or at 2-year post transplantation and before transplantation. We considered the participant’s phenotype 2 years after the sampling, I.e. at 3 years post transplantation for the one-year sample and at 4 years post transplantation for the two years sample. At the time of the sampling, all patients included were Stable

# Figure S2. Comparison of MMP-9 blood levels of Y2 analysis according to CLAD phenotypes.

p-value corresponds to comparison of MMP-9 value between CLAD type (BOS, RAS or mixed) with Stable


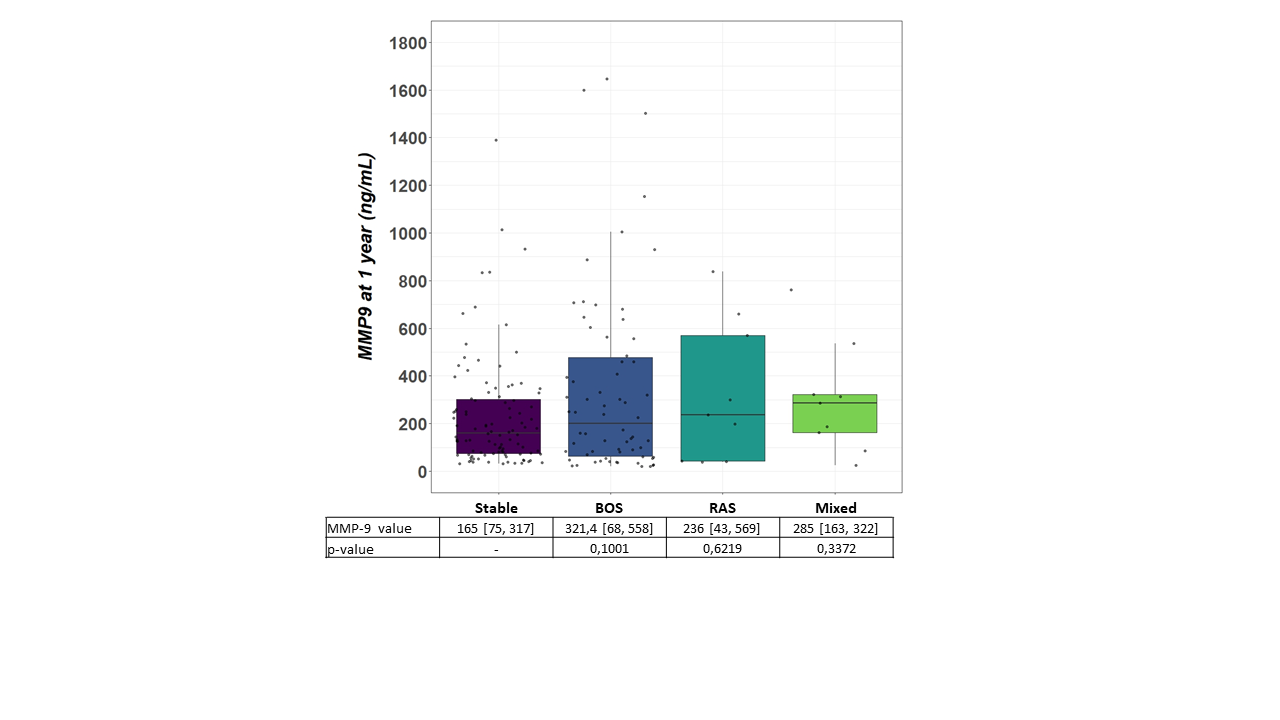


# Figure S3. Precision-Recall (A) and ROC (B) curves for the Y1 MMP-9 analysis

A


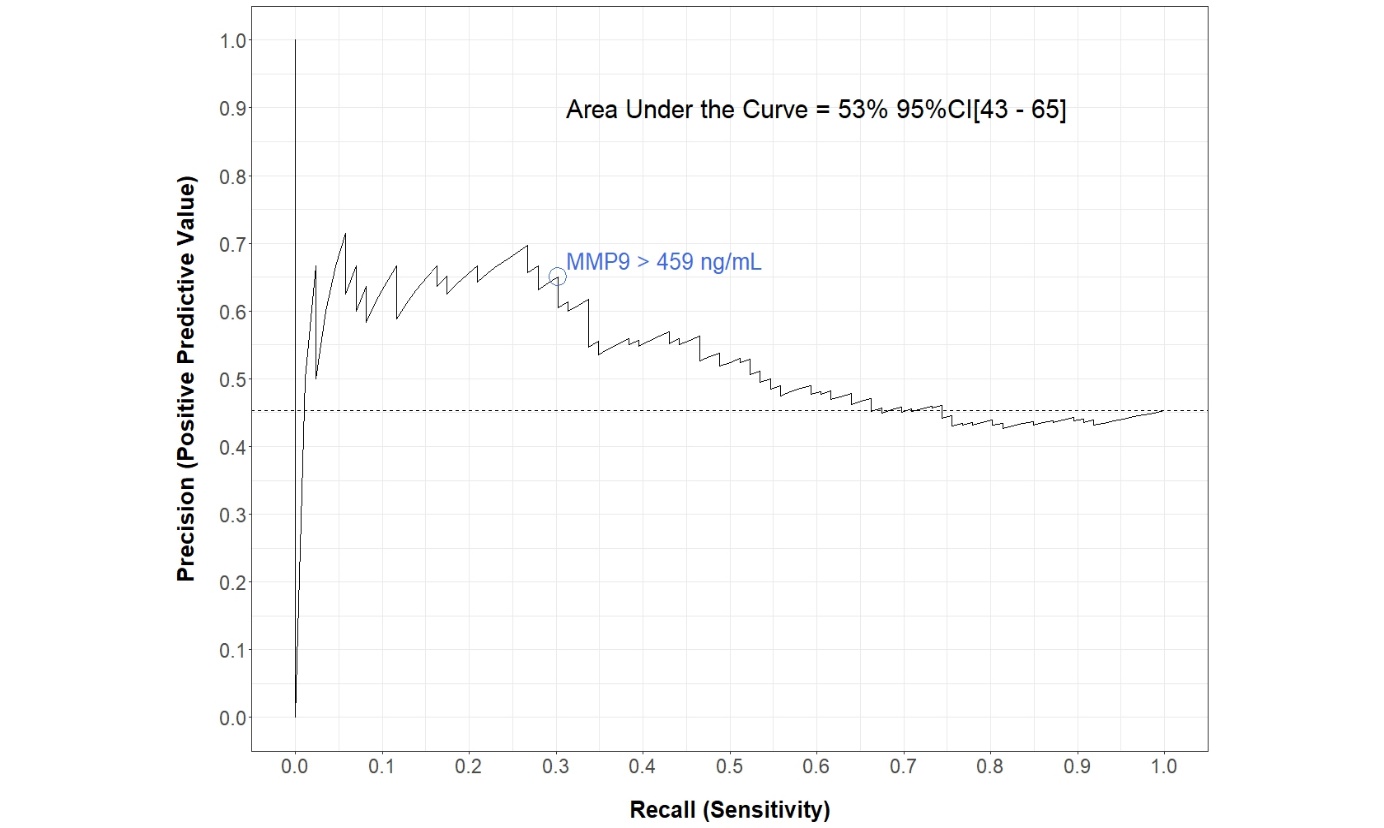


B

**
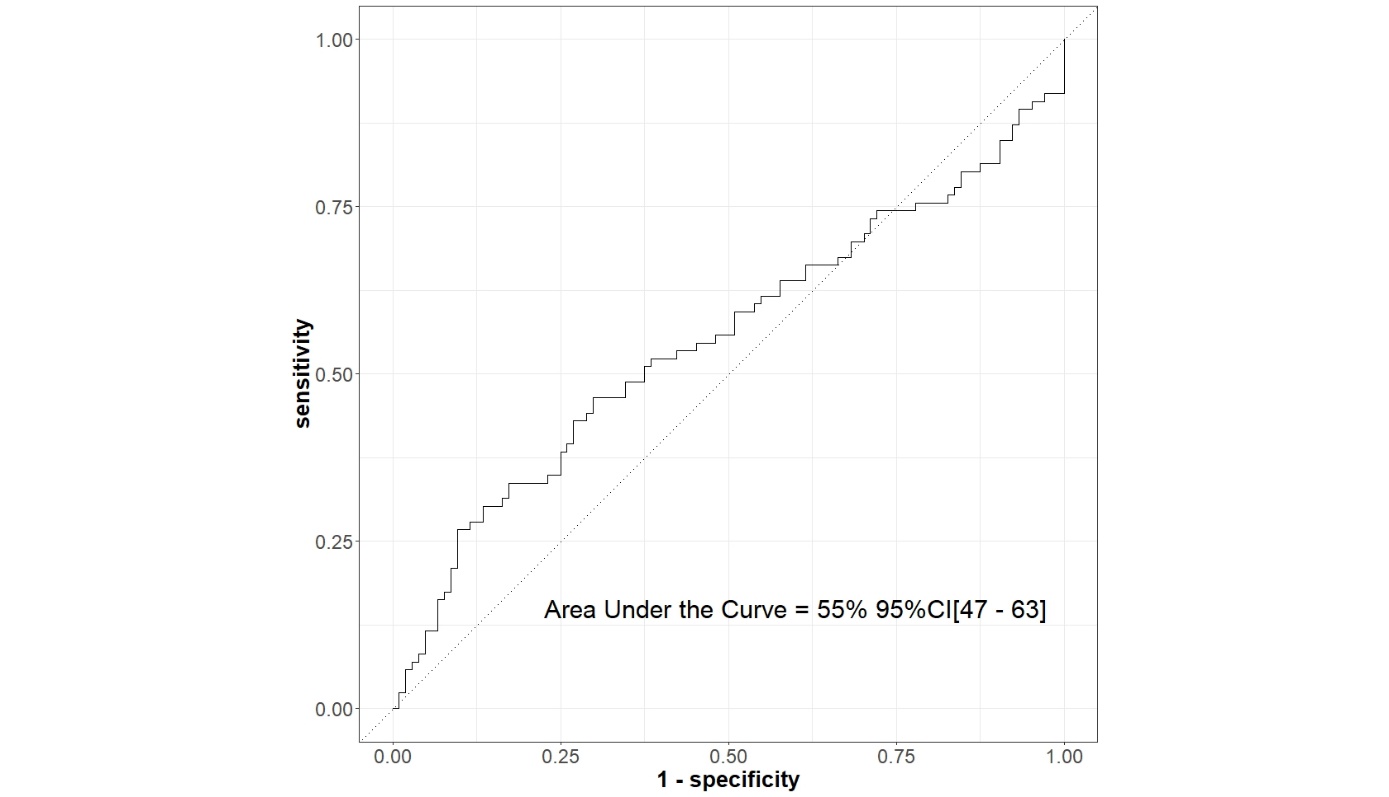
**

The precision-recall curve represents for each available value of MMP-9 its positive predictive value and ist sensitivity for CLAD onset within the next 2 years

# Figure S4. Comparison of MMP-9 blood levels of Y2 analysis according to CLAD phenotypes.

p-value corresponds to comparison of MMP-9 value between CLAD type (BOS, RAS or mixed) with Stable


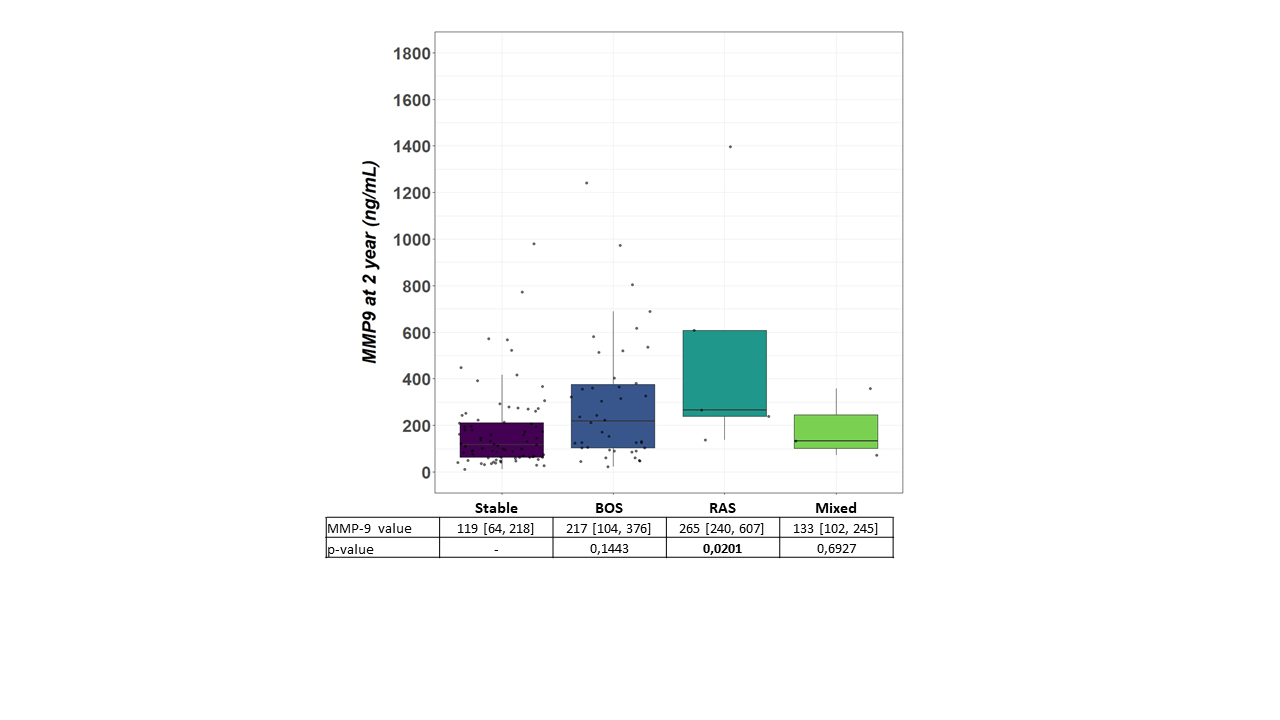


# Figure S5. Longitudinal analysis of MMP-9 blood levels for recipients with available samples before transplantation, at Y1 and Y2.

* Corresponds to p value < 0.05


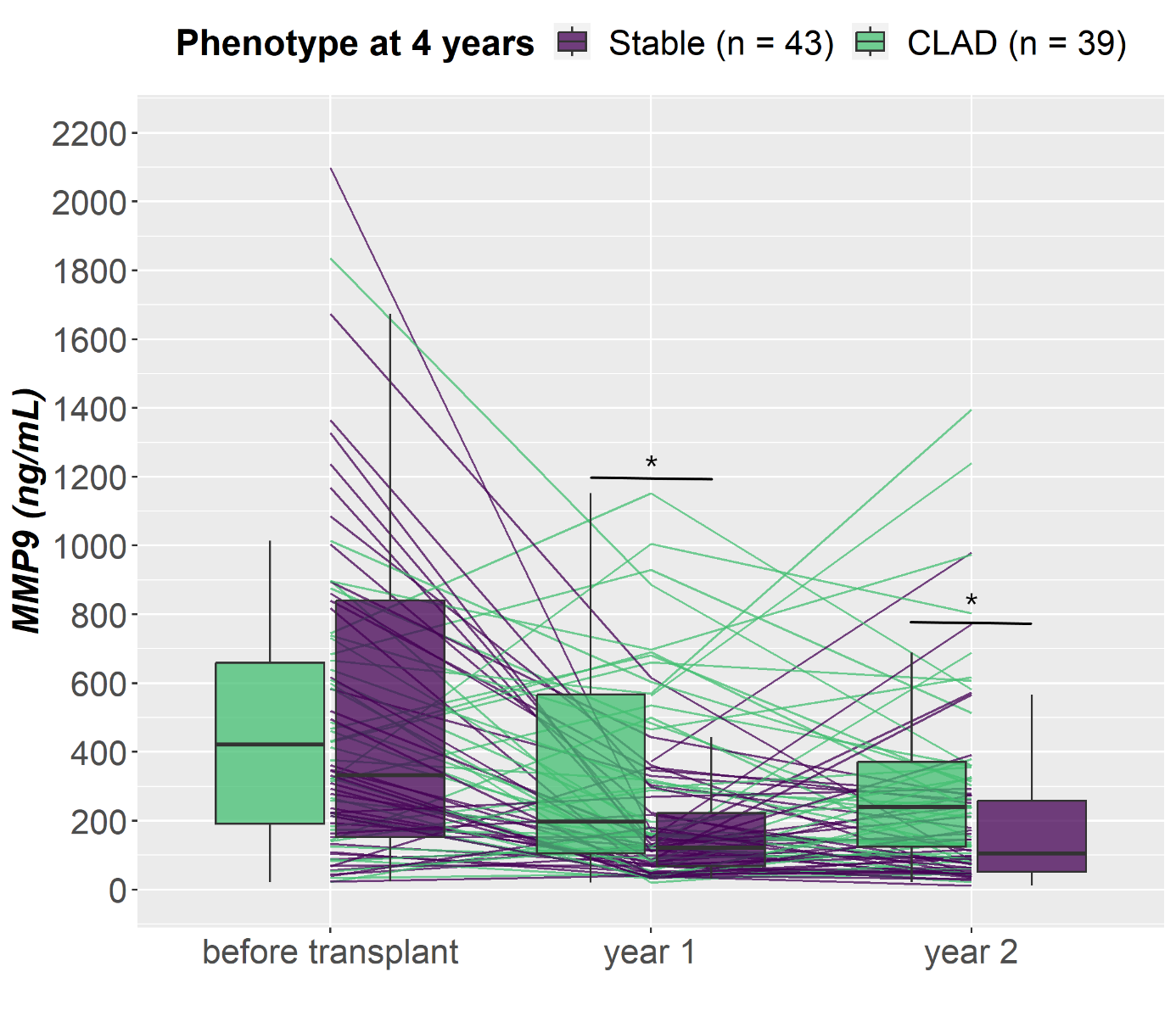

Supplement: Supplementary file 1 — Additional file 1: S1. COLT study protocol. S2. Description of variables of interest. Figure S1. Study protocol. Figure S2. Comparison of MMP-9 blood levels of Y2 analysis according to CLAD phenotypes. Figure S3. Precision-Recall (A) and ROC (B) curves for the Y1 MMP-9 analysis. Figure S4. Comparison of MMP-9 blood levels of Y2 analysis according to CLAD phenotypes. Figure S5. Longitudinal analysis of MMP-9 blood levels for recipients with available samples before transplantation, at Y1 and Y2. [file 12931_2024_2707_MOESM1_ESM.docx]
